# Healthcare inequities and healthcare providers: we are part of the problem

**DOI:** 10.1186/s12939-025-02464-9

**Published:** 2025-04-07

**Authors:** Crystal N. Campbell

**Affiliations:** DNP Nurse Anesthesia Degree Program Betty Irene Moore School of Nursing, University of California Davis, 2570 48th Street, Sacramento, CA 95817 United States of America

**Keywords:** Health inequity, Implicit bias, Healthcare disparities, Patient outcomes, Bias mitigation, Diversity, Health policy

## Abstract

**Background:**

The United States (U.S.) spends the highest amount on healthcare globally, at $12,434 per capita, yet experiences poor health outcomes, including lower life expectancy and higher rates of preventable mortality. With a life expectancy of 76.4 years, the U.S. lags behind other high-income countries, which have an average of 81.1 years. Health inequities, especially among marginalized racial and ethnic groups, contribute significantly to these disparities. Implicit bias among healthcare providers plays a critical role in perpetuating these inequities, resulting in misdiagnoses, undertreatment, and patient mistrust.

**Purpose:**

This paper examines the role of implicit bias in healthcare disparities, its impact on marginalized populations, and the ethical responsibility of healthcare providers in mitigating bias. It explores the neuroscientific and psychosocial mechanisms of implicit bias and its effects on patient outcomes.

**Methods:**

A literature review was conducted using PubMed, APA PsycNet, JSTOR, ProQuest, and Google Scholar. The search included peer-reviewed articles from 2008 to 2025 discussing implicit bias in healthcare, its effects on marginalized groups, and evidence-based mitigation strategies. Exclusion criteria included responses and commentaries.

**Findings:**

Quantitative findings on implicit bias mitigation strategies show mixed results. Counter-stereotypic strategies and intention-setting interventions reduced Implicit Association Test (IAT) scores by 0.15 at 4 weeks and 0.17 at 8 weeks. However, some strategies, like stereotype replacement and intergroup contact, consistently showed measurable positive effects. Qualitative findings revealed that simulation-based training and perspective-taking significantly increased self-awareness, empathy, and behavioral changes in healthcare providers. Mindfulness meditation and emotional regulation techniques helped reduce stress and bias in high-pressure settings. These findings suggest that while some strategies are effective in the short term, long-term success requires ongoing training, continuous reflection, and practical application in clinical practice.

**Conclusion:**

Health inequities in the U.S. are a public health crisis, disproportionately affecting marginalized groups. These disparities are preventable, yet persistent due to systemic issues. Healthcare providers must address implicit biases and commit to unbiased, ethical care. Institutions must prioritize health equity through inclusive cultures, comprehensive bias training, and accountability, exemplified by efforts like UW Medicine’s bias incident reporting.

**Supplementary Information:**

The online version contains supplementary material available at 10.1186/s12939-025-02464-9.

*“The eye sees only what the mind is prepared to comprehend.”*

*-Robertson Davies*

## Introduction

The United States of America spent 4.5 trillion dollars in healthcare expenditures in 2023 [1]. Compared to other developed countries, the United States (U.S.) spent the most on healthcare expenditures, at $12,434 per capita, with the average developing country per capita was $5,747 [2] (See Supplementary Table 1, WHO Global Health Expenditure Data Health Expenditures 2022 Developed Countries). Despite these drastic expenditures on healthcare, the United States has one of the lowest life expectancies at 76.4 years of age compared to other high-income countries, with an average life expectancy of 81.1 years of age [3, 4]. U.S. citizens are more likely to die at a younger age from avoidable causes, have the highest maternal and infant mortality, and are amongst those countries reporting the highest suicide rates [3]. An analysis conducted by LaVeist revealed that health inequities of under-represented ethnic and racial groups resulted in $421 billion in spending in the U.S [5]. Importantly, these rates reflect vast health inequities, defined by Wyatt as “health outcomes that are systematic, avoidable, and unjust” [6].

The United States healthcare system is in dire need of change. Given how the United States leads in many ways, can it ameliorate avoidable and unfair health disparities? The answer to bolstering the U.S. healthcare systems is not a simple solution as it encompasses a complex web of multiple stakeholders, shortages of healthcare professionals, increasing healthcare costs, lack of continuity of care, multifaceted insurance payer systems, delayed care, political health policy discord, lack of healthcare transparency, and a mirage of other factors. However, one intervention would help alleviate many U.S. patients’ health ailments and is within the reach of every healthcare professional. The answer is buried in the subconscious of all healthcare providers.

## Literature review

### Neuroscience and psychosocial aspects of implicit bias

Sigmund Freud suggested that the unconscious mind has a powerful influence on behavior [7]. The human body is a miraculous creation that can self-regulate without utilizing much brain power or exerting a great effort of energy. Many daily bodily tasks, such as processes involving the sympathetic and parasympathetic pathways, are automatic and require little concentration or energy. Part of the biological automatic processes in the brain is the development of thoughts and behaviors.

Unconscious (or implicit bias) results from social cues, activating the amygdala —an area in the brain involved in emotional processing [8]. A study by Sato demonstrated that the amygdala engages in emotional processing through subcortical pathways—deeper brain structures—prior to conscious awareness of the stimuli [9]. Specifically, the amygdala quickly processes what we see and automatically sorts people into groups based on categories such as gender, race, ethnicity, age, and sexual orientation, among others [10]. Accordingly, the amygdala unconsciously assigns attitudes or beliefs to these specific groups, which don’t necessarily align with explicitly (or consciously) held beliefs or values [8]. Implicit bias develops from unconscious negative and positive beliefs about social groups [7, 11]. These beliefs often serve as the foundation for stereotypes, prejudice, and discrimination [12]. All humans have bias as it’s a naturally ingrained primitive psychosocial process that allows us to quickly decipher a friend versus a foe in everyday interactions [11].

Although healthcare providers often strive to provide quality care to the patients they serve, biases and assumptions can impact the care they provide to various patient populations. Implicit bias operates unconsciously and is not rooted in malicious intent; however, it is essential to acknowledge that it can lead to significant harm [8].

### Social groups effected by implicit bias

This literature review demonstrated that implicit bias can be directed toward a range of social groups. Notably, a significant correlation has been identified between high levels of healthcare professional’s implicit bias and negative patient experiences [13]. Consequently, reports of discrimination by study participants are interpreted as manifestations of implicit bias. Throughout this section, references to discrimination are understood to reflect experiences rooted in implicit bias. The following sections will examine the social groups most prominently identified in the literature as being disproportionately affected by such discrimination.

#### Race/ethnicity

The literature revealed a widespread presence of discrimination against multiple racial and ethnic groups [7, 12–19]. From the studies gathered in this literature review, the ethnic and racial groups studied and identified as having experienced discrimination were African Americans, Asians, and Hispanics [12–17, 19]. However, these racial and ethnic groups are not an exhaustive list of groups experiencing discrimination. In Nong’s study, they found that discrimination against racial or ethnic groups was the most common type of discrimination [17]. Additionally, African Americans were found to experience discrimination the most of all races or ethnicities studied [17]. These findings underscore the persistent influence of structural racism and implicit bias in healthcare settings.

#### Gender

Gender inequality has long been a universal societal issue. Implicit gender bias was well-documented in the literature [12, 17, 20, 21]. Medeirt’s scoping review revealed that 9 out of 11 studies indicated a presence of gender bias against females within the clinical setting [21]. The implicit biases identified in these studies demonstrated an association between women and negative stereotypes, including perceptions of increased risk-taking behavior [21].

#### Age

Age-related implicit bias is prevalent, particularly toward elderly patients [17, 18]. A study conducted by Rogers involving participants 50 years of age and older revealed that age was the most commonly reported reason for experiencing discrimination in the healthcare setting [18]. Research revealed bias in perceptions of older patient’s pain perception and decreased support for oncology treatment in aging patients [21].

#### Sexual orientation and gender identity (LGBTQIA+)

The term “LGBTQIA+” encompasses individuals who identify as lesbian, gay, bisexual, transgender, quer or questioning, intersex, asexual, and plus, which includes people who identify by the terms “two-spirit,” “non-binary,” and “pansexual” [22]. Studies have revealed biases towards members of this community [7, 16]. However, compared to other socially marginalized groups, there remains a lack of research on bias against LGBTQIA + individuals in the healthcare setting [21]. Further research is needed to better understand and address the unique healthcare needs and disparities experienced by this population.

#### Religion

Religion can often be a politicized topic, which prevents patients from feeling comfortable disclosing their religious preferences. Since the attack on the United States on September 11th, 2001, many Muslims have experienced adverse reactions in the form of societal discrimination in regard to their religion [23]. Bias against religion was evident in the literature search [17, 23]. Although religious bias is not the most common form of implicit bias identified, its existence is not only evident but harmful, as religious bias can significantly impact patient-provider interactions, treatment decisions, and the overall quality of care, particularly when providers hold unconscious assumptions about patients’ beliefs, practices, or values [23].

#### Medical condition

The literature search revealed that certain medical conditions were associated with implicit bias. Obesity emerged as one of the primary medical conditions that was associated with bias in the clinical setting [21]. In one study, healthcare practitioners were found to have negative implicit bias toward obese patients [24]. Another study reported that some providers attributed obesity to poor self-control and lacking personal responsibility despite evidence that obesity is a complex condition influenced by genetics, access to affordable healthy food, stress, and epigenetic mechanisms [25, 26]. These beliefs, rooted more in opinion and negative attitudes than in scientific evidence, are problematic. Obese patients often present with multiple comorbidities and require frequent healthcare interactions and interventions, making equitable, evidence-based care essential. It is, therefore, critical for healthcare providers to receive training in evidence-based obesity healthcare management to better support patients in achieving optimal health outcomes without judgment or stigma. Additionally, Human immunodeficiency virus (HIV), a stigmatized disease, was identified as another medical condition that was associated with implicit bias in healthcare settings [7].

#### Physical disabilities

The Americans with Disabilities Act of 1990 mandates equal access to healthcare services and facilities [27]. However, implicit bias against patients with disabilities has been demonstrated in the literature [7, 18]. In one study, researchers posed as caregivers for a fictitious patient with disabilities and contacted various facilities; many denied care or claimed they could not accommodate the patient based on their history [27]. Although healthcare facilities are required to make accommodations for patients with special needs, these facilities denied care based on the patient’s history of physical disability. This reflects a troubling disconnect between legal requirements and real-world practice, highlighting how prejudice and misinformation can override regulatory compliance. Such bias not only violates civil rights but also contributes to health disparities by limiting access to timely and appropriate care.

#### Mental illness

Implicit bias towards individuals with mental illness, including those with substance use disorders, was identified in the literature [17, 28]. One study focusing on Assertive Community Treatment (ACT) providers for mental health patients revealed that many of the ACT providers had negative explicit and implicit biases, which involved beliefs that mental illness patients were dangerous, helpless, and blameworthy [28]. These biases can have serious consequences in clinical settings, influencing the quality of care provided and potentially leading to discriminatory treatment practices. For example, providers may be less likely to engage empathetically with patients or may underestimate their capacity for recovery and autonomy. Such attitudes can undermine therapeutic relationships and discourage individuals from seeking or continuing care.

#### Socioeconomic factors (education and income level)

Socioeconomic factors within the U.S. patient population serve as predictive measures of healthcare outcomes [29]. Moreover, these factors can contribute to the formation of implicit bias among healthcare providers. For example, implicit bias related to educational attainment was documented in the literature [17]. Additionally, patients with incomes lower than $50,000 annually were more likely to experience discrimination in healthcare settings [17]. These findings suggest that perceptions of socioeconomic status can shape provider assumptions about a patient’s health literacy, compliance, and lifestyle choices. Such beliefs may influence clinical decision-making, potentially leading to inadequate communication, reduced patient engagement, or fewer referrals for specialized care. This contributes to a cycle in which lower-income patients receive suboptimal care, exacerbating existing health disparities.

Healthcare providers must acknowledge the prevalence of implicit bias, particularly its disproportionate impact on specific populations identified in the literature. These socially marginalized groups have consistently reported experiencing significant discrimination within healthcare settings. To ensure equitable and high-quality care, it is essential that providers engage in critical self-reflection to examine their own biases towards these patient populations, ensuring they provide the best care possible. Increasing awareness of one’s implicit bias remains an essential strategy in mitigating it [8].

### Healthcare professionals and implicit bias

Healthcare professionals spend a significant portion of their careers working with people. They encounter people across the spectrum of socioeconomic statuses, racial and ethnic backgrounds, and ages. Studies have revealed that healthcare professionals exhibit implicit bias at the same rate as the general population [30]. Among the groups affected, black people experience the highest prevalence of discrimination in healthcare settings [15].

But to what extent does implicit bias among healthcare providers contribute to health inequities in the United States? Research has demonstrated that implicit bias has contributed to a mirage of negative consequences for patients, influencing clinical decision-making, treatment recommendations, and overall quality of care [7, 13, 31–34]. A growing body of literature reinforces the connection between discrimination and adverse health outcomes for marginalized groups in the United States [15, 19, 31, 34, 35].

### Implicit bias effects on health outcomes

#### Direct effects of implicit bias

Research revealed widespread disparities in care quality due to discrimination. The Agency for Healthcare Research and Quality (AHRQ) published a report that concluded that white patients received better quality of care than patients who were Black, Latino, Asian, Native American, Alaska Native, Native Hawaiian, and Pacific Islander [36]. Furthermore, studies support the notion that implicit bias influences clinical decision-making. A literature scoping review by Meidert found that healthcare providers often rely on learned stereotypes about a patient’s physical characteristics, which, in turn, shape their expectations [21]. For example, providers tend to associate certain diseases with specific racial or ethnic backgrounds [21]. This impacts clinical decisions, leading to diagnostic errors, inappropriate treatment recommendations, and prescribed pharmacological treatments, which ultimately result in disparities in care [21, 33]. The Institute of Medicine’s (IOM) report, “Ending Unequal Treatment,” also supported the notion that implicit bias contributes to the misdiagnosis and undertreatment of patients from under-represented groups [31]. The National Cancer Institute (NCI) reported that patients from under-represented groups have a higher incidence, mortality, and more advanced staging at the time of diagnosis for cancers, including cervical, kidney, breast, colorectal, lung, and prostate cancer [37]. These disparities stem from inadequate screening practices and inappropriately prescribed treatment plans by healthcare providers [37]. One study indicated that those who experienced an event of discrimination in the healthcare setting were more likely to have a new or worsened disability within the subsequent 4 years [18].

Although studies by the IOM, NCI, AHRQ, and others have documented misdiagnosis, undertreatment, lower quality of care, increased incidence of new or worsening disability, and advanced-stage cancer diagnoses among underrepresented populations, a scoping review by Meidert, which synthesized findings from 81 studies published over the past decade, did not identify a consistent correlation between these factors and adverse patient outcomes [21]. These findings suggest that the relationship between implicit bias and health disparities may be more nuanced and less uniformly pervasive than previously assumed.

#### Indirect effects of implicit bias

Implicit bias can indirectly impact patient care outcomes through the patient’s perception of discrimination, as influenced by communication and nonverbal interactions [32]. Though they are not conscious of it, healthcare provider’s implicit bias affects non-verbal behavior and communication in the presence of patients [13]. Patients not only listen to what providers say but also how they say it through tone, pitch, and affect [38].

Implicit bias can manifest in subtle yet impactful ways, such as indications of uneasiness or behaviors that suggest discomfort or dislike. These include a lack of eye contact, physical proximity to the patient, body language, and speech errors [13, 38]. In addition, dominating the conversation during interactions, micro-aggressive comments (or subtle comments with underlying negative connotations), and excluding the patient’s input in their plan of care are indicators of implicit bias [39, 40].

Perceived discrimination during clinical encounters has been linked to a decline in trust in healthcare providers and a decrease in satisfaction with care [41]. As a result, patients may become reluctant to seek medical services and may struggle with adherence to treatment plans, increasing the risk of disease progression and poor health outcomes [14, 30, 41]. A study supporting this conclusion found that Black and Latino elderly patients who perceived experiencing discrimination and receiving substandard care had an increased likelihood of developing dementia later in life [42].

Further research into implicit bias within the U.S. healthcare system revealed that it is shaped by structural racism [43]. The racial struggles of the United States − marked by legalized segregation and systemic discrimination against African Americans − have left lasting effects [31]. While laws have been enacted to combat segregation and discrimination, the consequences of this highly racialized history persist today, including the healthcare setting [31].

Underrepresented racial groups experience persistent health inequities, driven in large part by endemic stress − prolonged, repetitive stress embedded in daily life experiences − by ways of enduring racism [44]. This continuous exposure to stress, compounded by systemic inequities within healthcare, serves as a root cause of health disparities [43]. Therefore, the development of illness is not solely attributable to natural biological factors or inherent disease processes but is increasingly understood to be shaped by experiences of discrimination and systemic inequities.

Discrimination can have direct physiological and psychological consequences, contributing to poor health outcomes. Victims are more likely to develop anxiety and depression, experience heightened stress levels, and engage in behaviors such as increased alcohol consumption − all of which accelerate aging and negatively impact health [7, 15, 40, 45–47]. Current research has focused on the impact of environmental stressors, such as discrimination, on the healthcare outcomes of African Americans. Simons et al. established a connection between race-related stressors experienced during adolescence in African Americans and increased inflammation in adulthood [48]. Increases in inflammation of the body increase the prevalence of various diseases such as cardiovascular disease, type II diabetes, autoimmunity, gastrointestinal disorders, respiratory disease, and neurogenerative diseases, among many others [49].

While healthcare providers cannot protect under-represented populations from the everyday discrimination encountered outside clinical settings, they have a critical role in minimizing such experiences within healthcare environments by actively addressing their own implicit biases. This effort requires the implementation of implicit bias mitigation strategies, which will be discussed in the following section. Furthermore, providers should be educated on the prevalence and impact of everyday discrimination on health outcomes among marginalized groups. During clinical encounters, healthcare professionals should assess patients’ psychosocial needs, evaluate current or historical mental health concerns, promote wellness strategies, and provide appropriate resources to support optimal health outcomes.

### Mitigation strategies for implicit bias

How can a healthcare provider address implicit bias if they are not even aware of it? This is one of the most challenging aspects of mitigating implicit bias. Many individuals believe they uphold egalitarian values and reject prejudice, yet they may still harbor deeply ingrained biases. However, all humans have biases [11].

Howard Ross, in *Everyday Bias: Identifying and Navigating Unconscious Judgements In Our Lives* explains this paradox: “Ironically, on an unconscious level, somebody (even of color) who sees himself as liberal on racial issues, for example, may have unconscious biases that are not much different from those possessed as an overt [explicit] racist” [11]. This highlights the importance of self-awareness and acknowledgment. Healthcare providers, like all individuals, must first accept that they have implicit biases before they can work toward mitigating them.

Research has revealed that healthcare participants of studies have reported low levels of explicit bias while exhibiting high implicit bias towards specific social groups [50]. This discrepancy can create significant challenges in healthcare settings. When individuals believe they are free of bias, they may struggle to recognize the necessity of implicit bias training, potentially hindering efforts to improve equitable patient care. Even if it is mandated to attend implicit bias training, it is not consistently developed based on evidence-based interventions that effectively bring about self-actualization, thought, and behavior change [51]. This reinforces the public perception that implicit bias training is unnecessary.

Poorly designed Diversity, Equity, and Inclusion (DEI) initiatives—particularly those that implement implicit bias training without incorporating evidence-based interventions—can be counterproductive. When companies fail to see meaningful outcomes, these programs are often viewed as ineffective and a waste of resources. Major corporations, such as Walmart, Ford Motor Company, Harley Davidson, John Deere, Caterpillar, Tractor Supply, and Lowe’s, have scaled back their DEI training efforts [52]. However, in the healthcare sector, the consequences of abandoning DEI education are far more significant. Comprehensive training that incorporates both implicit bias education and evidence-based mitigation strategies is essential, as unaddressed implicit bias contributes significantly to profound health inequities in the U.S [53].

Many studies have been conducted on effective mitigation strategies to break the cycle of automatic biased thoughts. Before discussing these strategies, the literature has suggested specific guidelines and recommendations for structuring implicit bias mitigation training. Evidence indicates that the process involves habit-breaking strategies involving a continuous process that requires effort and motivation [54]. A “one and done” training is insufficient to drive significant and long-term changes in individuals [54]. Furthermore, “normalizing” implicit bias as a common and natural occurrence creates a safe space for participants to speak openly and honestly about bias without shame and judgment [55, 56].

Participants must first acknowledge that bias exists while understanding the negative impact on patients [57]. They must openly acknowledge and take ownership of personal stereotypes and prejudice, which is essential in mitigating implicit bias [12, 55]. Additionally, participants must go a step further by taking responsibility for correcting these beliefs by seeking educational opportunities to adequately and meaningfully abate these biased thoughts.

Educators of these training programs should recognize that motivation is a key factor in facilitating meaningful change. Participants who strive to be unbiased and express concern about the adverse effects of bias are more likely to be open to implicit bias training [55, 58]. Furthermore, educators must warn participants that high-stress situations and negative emotions can increase implicit bias toward patients of certain social groups [59]. Providers should be more vigilant in their interactions in clinical environments that require stressful, time-sensitive, and high-stakes decisions, such as emergency department and anesthesia healthcare providers [52].

The foundation of implicit bias training should start with providing information. In a study by Sims, the authors conducted a systematic review that identified the most effective strategies for incorporating implicit bias into an educational program for health professionals. One of their recommendations was to educate participants on the science of implicit bias, covering its different forms—implicit versus explicit and positive versus negative—as well as the psychosocial and neuroscientific mechanisms underlying it, as previously discussed [60].

After building a foundational understanding of implicit bias, individuals must begin the process by recognizing their own implicit biases. This can effectively be obtained by taking an Implicit Association Test via Project Implicit from Harvard University [61]. These tests have been widely used in research studies on implicit bias and have proven to be a reliable tool for revealing implicit bias. Once individuals have acknowledged their implicit bias, they can access and practice mitigation strategies to help mitigate it.

There are a variety of implicit bias mitigation strategies named in the literature. These include continuous reflection on one’s beliefs on specific social groups, gaining access to regular training, and seeking feedback from outside sources such as the IAT [8]. In a comprehensive review of the literature, Azman concluded that educational sessions combined with hands-on practice of bias mitigation strategies proved helpful for participants [62]. Vora states, “Simulation-based education provides an opportunity to promote changes in knowledge, skills, attitudes, and behaviors through the deliberate practice of IBMS [implicit bias mitigation strategies] and self-reflective debriefing” [63]. Other strategies identified included stereotype replacement, counter-stereotypic imaging, individuation, perspective-taking, intergroup contact, partnership building, emotional regulation, mindfulness meditation, and the use of evaluative conditioning [13, 50, 56, 58, 64, 65]. Descriptions of each strategy are provided in Table 1. 

**Table 1:** Descriptions of Mitigation Strategies

Mitigation strategy	Description
Self-reflection	Increase self-awareness by taking Harvard’s Implicit Association Test (IAT) and reflecting on revealed biases. Make active efforts to combat these specific biases through self-monitoring/self-regulation [8,60].
Seeking feedback	While participating in simulation-based implicit bias mitigation training, an outside perspective can help one identify underlying biases they would not otherwise be aware of [8].
Hands-on/Simulation-based education	Creating a safe environment where participants can participate in scenarios provides an opportunity for participants to elicit changes in knowledge, skills, attitudes, and behavior [62].
Stereotype replacement	Acknowledging that one’s response is influenced by a stereotype and making a deliberate effort to adjust it [58].
Counter-stereotypic imaging	Imagining a person associated with a specific social group as being the opposite of a stereotype of that group [58].
Individuation	Focusing on a person as an individual set apart from their social group and any stereotypes associated with that social group [58].
Perspective taking	“Putting yourself in another person’s shoes” increases empathy and concern for other social groups [58].
Intergroup contact	Immersion in another culture/social group reduces prejudice and reduces healthcare provider anxiety while boosting their confidence during interpersonal communication [65].
Partnership building	Healthcare providers redefining their relationships with their patients as more of a partnership where patients collaborate with their providers when deciding on a healthcare plan instead of resembling a one-sided authoritative role of the practitioner [58].
Emotional regulation	Reducing anxiety in stressful environments reduces implicit bias [55].
Mindfulness meditation	Engaging in meditative practices, including yoga, results in less implicit bias in the clinical setting [71].
Evaluative conditioning	Associating specific social groups with positive attributes [72].

One frequently cited study in the implicit bias literature is a 2012 study conducted by Devine, which was the first of its kind to demonstrate the effectiveness of mitigation strategies. In the study, participants completed an IAT, received feedback on their results, and reviewed educational materials discussing implicit bias—including how it can lead to discrimination—and various mitigation strategies [58]. These strategies included stereotype replacement, counter-stereotypic imaging, individuation, perspective-taking, and intergroup contact, along with guidance on how to apply each technique [58]. Participants were also asked to generate examples of how they might implement each strategy in real-life situations [58]. The intervention group underwent follow-up assessments at four and eight weeks after the initial intervention [58]. Results from these assessments showed a consistent reduction in IAT D-scores by 0.15 at 4 weeks and 0.17 at 8 weeks [58]. These findings suggest that the intervention resulted in a sustained reduction in implicit bias, lasting at least two months.

While some studies show promising results for various implicit bias mitigation strategies, others highlight their limitations. A 2022 literature review by Greenwald found that, with the exception of counter-stereotypic strategies and intention-setting, most approaches lack proven effectiveness [56]. Similarly, Forscher reported that although their interventions — including taking an IAT and prejudice habit-breaking interventions — increased participants’ concern about bias, they did not decrease implicit bias compared to the control group [66]. These findings suggest that not all strategies are equally effective.

Given the mixed findings in the literature, how should organizations determine which interventions to include in implicit bias training? During the Scientific Workforce Diversity Seminar Series (SWDSS), expert panelists–who were scholars of diversity and implicit bias training–gathered to discuss their recommendations. They advised organizations to set clear Diversity, Equity, Inclusion, and Accessibility (DEIA) goals supported by evaluative tools [67]. Trainings should prioritize the most effective evidenced-based implicit bias mitigation strategies that promote positive approaches and messaging, model appropriate behavior, are easy to implement, and include skill-building exercises that allow participants the opportunity to practice mitigation strategies in a practical engaging manner [67]. By adopting a strategic, evidence-based approach, organizations can amplify the impact of implicit bias training and foster meaningful, sustainable change within the workplace.

## Conclusion

Health inequities among marginalized populations in the United States have been extensively studied and recognized as a public health crisis by leading organizations, including the World Health Organization (WHO), the Agency for Healthcare Research and Quality (AHRQ), and the National Institutes of Health (NIH). These inequities, defined as avoidable and unjust differences in health outcomes among social groups, impose significant economic and societal burdens—yet they remain preventable [6].

Healthcare providers play a vital role in addressing health inequities, as implicit biases can unintentionally influence clinical decisions and patient interactions. By recognizing and actively mitigating these biases, providers can help ensure more equitable care and outcomes. Upholding the ethical principle of non-maleficence requires a commitment to fair and unbiased treatment. Providers must acknowledge their potential role in perpetuating disparities and take responsibility for being part of the solution.

Healthcare institutions have a critical responsibility to make health equity a strategic priority of the organization [68]. Organizational leadership should foster a culture that is progressive, inclusive and equitable to reduce health disparities and ultimately address health inequities affecting under-represented social groups [69]. One key strategy is to provide clinicians with opportunities for training on implicit bias. Additionally, there should be an ongoing evaluation of practitioner progress to ensure accountability. Monitoring practitioner behavior outcomes helps maintain standards of equitable care. For example, UW Medicine publishes a quarterly report detailing the number of bias-related incidents, serving as a model of transparency and accountability [70].

To truly embed equity within healthcare systems, these initiatives must be sustained and integrated into the organization’s core operations. Equity should not be treated as a one-time project but as a continuous commitment woven into clinical practice, policies, and institutional values. Only through intentional and consistent efforts can healthcare systems ensure that all patients—regardless of race, gender, income, or ability—receive fair and compassionate care.

## Electronic supplementary material

Below is the link to the electronic supplementary material.

Supplementary Material 1: WHO Global Health Expenditure Data Health Expenditures 2022 Developed Countries

## Data Availability

No datasets were generated or analysed during the current study.

## Declarations

### Author’s information

C.N.C. is a Health Sciences Clinical Assistant Professor at the University of California Davis at the Betty Irene Moore School of Nursing for the Doctor of Nursing Practice Nurse Anesthesia Degree Program. She is a Certified Registered Nurse Anesthesiologist with a Doctorate in Nurse Anesthesia Practice from Texas Wesleyan University and a Master of Science in Anesthesiology from Barry University. She has 14 years of practice in the field of anesthesia, serving in the Florida and Minnesota communities. Additionally, she is an active American Association of Nurse Anesthesiology (AANA) member and serves on the Diversity, Equity, and Inclusion Committee. She is an active member of the Florida Association of Nurse Anesthesiology (FANA) and serves on the Diversity, Equity, and Inclusion and Government Relations Committees.
